# Competing Risk Survival Analysis of Time to In‐Hospital Recovery or Death Among COVID‐19 Patients: A Hospital‐Based Multicenter Retrospective Cohort Study in Southeast Ethiopia

**DOI:** 10.1002/hsr2.72191

**Published:** 2026-03-24

**Authors:** Addis Wordofa, Ayalneh Demissie, Abdurehman Kalu, Abdurehman Tune, Mohammed Suleiman Obsa, Abay Kibret, Zerihun Abera, Yonas Mulugeta

**Affiliations:** ^1^ Department of Public Health College of Health Science, Arsi University Asella Ethiopia; ^2^ Department of Anesthesia College of Health Science, Arsi University Asella Ethiopia; ^3^ Department of Nursing College of Health Science, Arsi University Asella Ethiopia; ^4^ Department of Biomedical College of Health Science, Arsi University Asella Ethiopia

**Keywords:** competing risks, COVID‐19, Ethiopia, Fine‐Gray model, in‐hospital mortality, prognostic score, risk stratification, survival analysis

## Abstract

**Background and Aims:**

In‐hospital death and recovery are competing risks in COVID‐19 patients, complicating prognosis. While international prognostic scores exist, their reliance on complex biomarkers limits their utility in resource‐constrained settings. This study aimed to estimate the duration and identify determinants of in‐hospital outcomes in southeast Ethiopia and to develop a clinically accessible risk stratification tool.

**Methods:**

Data from 827 confirmed COVID‐19 patients (October 2022–May 2023) across six treatment centers were analyzed using the Fine‐Gray Competing Risk Survival Analysis (CRSA). Additionally, a novel “Asella COVID‐19 Risk Score” was developed using readily available bedside clinical parameters (age, comorbidity, and antibiotic use) and validated using receiver operating characteristic analysis.

**Results:**

Overall, 139/827 (17%) died, and 516/827 (62%) recovered. Risk of death was significantly higher for patients aged ≥ 50 years (acsHR = 2.62; 95%CI: 1.29, 5.29; *p* < 0.001) and those with an immunocompromised state (acsHR= 1.46; 95%CI: 1.08, 1.98; *p* = 0.014). Median time to death was 5 days. The “Asella COVID‐19 Risk Score” demonstrated an area under the curve of 0.65. At an optimal cutoff of ≥ 4, the score achieved a sensitivity of 47.2%, specificity of 73.2%, and a high negative predictive value (NPV) of 83.5%.

**Conclusion:**

Advanced age and immunocompromised status significantly increase mortality risk. The Asella COVID‐19 Risk Score provides a clinically validated, high‐NPV triage tool suitable for resource‐limited settings, facilitating the early identification of high‐risk patients and more efficient resource allocation.

**Abbreviations**
acsHR: adjusted cause‐specific hazard ratio
AUC: area under the curve
CI: confidence interval
CIF: cumulative incidence function
COVID‐GRAM: COVID‐19 Global Risk Assessment Model
CRSA: competing risk survival analysis
csHR: cause‐specific hazard ratio
IQR: inter quartile range
NPV: negative predictive value
ROC: receiver operating characteristic
RT‐PCR: Reverse Transcription Polymerase Chain Reaction
SARS‐CoV‐2: Severe Acute Respiratory Syndrome Coronavirus 2
SD: standard deviation
STROBE: Strengthening the Reporting of Observational Studies in Epidemiology

## 1 Introduction

The COVID‐19 pandemic, caused by SARS‐CoV‐2, has significantly impacted global human life and health systems [1, 2]. While morbidity and mortality rates varied across regions, Africa reported relatively fewer cases despite fragile healthcare infrastructures and large vulnerable populations [3, 4, 5, 6, 7]. Variations in disease burden were influenced not only by public health measures but also by demographic and clinical factors [8, 9, 10, 11].

Ethiopia, second to South Africa in Africa, experienced a high burden of COVID‐19 cases [9, 12]. The government implemented mitigation strategies, including testing, contact tracing, isolation, and mandatory quarantine [13, 14, 15]. Despite these interventions, the risk of in‐hospital mortality remained substantial due to comorbidities, delayed healthcare access, and resource limitations [16, 17, 18, 19].

The prognostic landscape for COVID‐19 has been shaped by the development of various clinical risk scores designed to assist in patient stratification. During the first and second waves of the pandemic, international tools such as the 4C Mortality Score and COVID‐GRAM demonstrated high discriminatory power in identifying patients at risk of severe outcomes [20, 21]. However, these models frequently rely on inflammatory biomarkers and laboratory data that may not be consistently available in resource‐limited clinical settings. Consequently, there is a pressing need for locally validated, clinically accessible tools that can provide rapid bedside triage in high‐burden, resource‐constrained environments.

For hospitalized COVID‐19 patients, outcomes are dichotomous: recovery or death. These events are competing risks, as the occurrence of one precludes the other. Traditional survival analyses, like Kaplan–Meier, may overestimate survival probabilities when competing events are present [22, 23]. The Competing Risk Survival Analysis (CRSA), particularly the Fine‐Gray model, allows accurate estimation of the cumulative incidence of competing outcomes [24, 25].

Therefore, this study aimed to analyze the time to in‐hospital COVID‐19 recovery or death using a competing‐risk survival approach and to identify independent determinants of mortality and recovery using CRSA. Understanding these predictors is crucial for early risk stratification and improved clinical management of hospitalized COVID‐19 patients in Ethiopia.

## 2 Methods

**Study Design:** A retrospective multicenter cohort study was conducted.

**Setting:** The study was conducted in six COVID‐19 isolation and treatment centers in southeast Ethiopia: Bishoftu, Modjo, Adama, Negele Arsi, Bekoji, and Shashamane. All confirmed COVID‐19 cases were admitted for monitoring and treatment during the study period [26].

### 2.1 Participants

All confirmed adult COVID‐19 patients admitted between October 1, 2022, and May 31, 2023, were **eligible** for the study. Patients were specifically **included** if they had a positive RT‐PCR result recorded after 24 h of hospitalization and possessed documented outcomes of either death or recovery. Records lacking essential data on demographics, outcome, or admission/discharge dates were **excluded**; this step led to the **exclusion** of 11 records out of the 838 initially **eligible** records (1.3%). Consequently, a total of **827** complete patient records were analyzed.

### 2.2 Variables

The study defined two outcomes: **Time from admission to death** (designated as Event 2, the competing event) or **recovery** (designated as Event 1, the primary event). The covariates included were **age, sex, location, comorbidities, clinical presentation, ventilatory support, and treatment modalities**. Age was treated as a continuous variable for descriptive statistics but was categorized as < 50 years versus ≥ 50 years in the multivariable model. The term **Comorbidity** was defined as the co‐existence of one or more pre‐existing conditions (≥ 1); critically, each of the specific comorbidities was treated as a separate variable in the multivariable analysis.

### 2.3 Data Sources/Measurement

Information was collected using structured data extraction sheets derived from clinical charts and hospital logbooks, following national COVID‐19 protocols [25, 27]. **Event 1 (Recovery)** was defined as clinical improvement leading to discharge, while **Event 2 (Competing Event)** was defined as in‐hospital death from COVID‐19. Censored cases included patients transferred to another facility, those lost to follow‐up, or patients still admitted at the end of the study. The **time‐to‐event** was uniformly calculated from the date of the positive COVID‐19 test to the date of the event.

### 2.4 Bias

Data quality was ensured through training of five public health officers as a data collector for 2‐days, real‐time supervision ensured completeness, and Selection and information biases were minimized via careful validation of records.

### 2.5 Sample Size

The initial sample size calculation yielded a required *N* of 419, which was derived assuming a survival probability (*p*) of 0.46, a 95% confidence level, a margin of error (*d*) of 0.05, and adjusting for a 10% withdrawal rate. To account for heterogeneity across the sampled hospitals, a **design effect** (Deff) of 2 was introduced, which doubled the target requirement to 838 (i.e., 419 × 2 = 838). After this determination, 11 incomplete patient records were excluded from the total collected data. Given the decision to include all remaining eligible records from the six study centers, the research ultimately utilized a final sample size of **827**.

### 2.6 Statistical Analysis

Data were analyzed using **STATA Version 16.1**. For descriptive statistics, Means ± Standard Deviations (SDs), medians (IQRs), and frequencies (%) were calculated [28]. Given the presence of mutually exclusive outcomes, a **Competing Risk Survival Analysis (CRSA)** was performed. The **Fine‐Gray model** was employed to estimate the cumulative incidence functions (CIFs) for both death and recovery [23, 25, 29, 30, 31, 32], and **Gray's test** was used to compare the CIFs between subgroups [29, 30, 31, 32]. In the multivariable CRSA model, variables with a *p*‐value of ≤0.25 in the bivariable analysis were included. Results are reported as adjusted sub‐distribution Hazard Ratios (acsHRs) with 95% Confidence Intervals (CIs). All tests were two‐sided, with statistical significance set at *p* < 0.05 [28]. Finally, a complete‐case analysis approach was applied to handle missing data. Furthermore, we developed the “Asella COVID‐19 Risk Score” to translate these multivariable findings into a practical bedside tool. We utilized β‐coefficients from our model to assign integer points to significant clinical predictors (age, comorbidity, and antibiotic use). The score's discriminative performance was validated using the Receiver Operating Characteristic (ROC) curve and area under the curve (AUC) analysis, with optimal cutoff points determined to maximize clinical utility for patient triage.

## 3 Results

### 3.1 Baseline Characteristics

**Demographic Summary:** The final sample size included **827 patients** with a mean age of 51 ± 18 years, with 55.2% of patients being ≥ 50 years old. Demographic representation showed that 62.5% (429/687) were male and 75.1% (601/800) resided in urban settings (Table 1).

**Table 1:** Demographic characteristics of adult COVID‐19 patients (*N* = 827 analyzed).

Variable	Category	*N* (Count)	% (Percentage)
**Age** (Total *N* = 688)			
	< 50 years	308	44.8
	≥ 50 years	380	55.2
**Sex** (Total *N* = 687)			
	Female	258	37.5
	Male	429	62.5
**Location** (Total *N* = 800)			
	Urban	601	75.1
	Rural	199	24.9

*Note:* Percentages may not sum to 100% due to rounding. The *N* value for individual variables is the number of records with non‐missing data for that variable.

**Clinical Presentation and Comorbidities:** Upon admission, 77% (637/827) of the cohort presented with clinical symptoms. Overall, **46.4% (384 patients)** had at least one comorbidity, with hypertension (31.5%), diabetes (28.7%), and an immunocompromised state (20.3%). A significant proportion of patients, 63% (522/827), required ventilatory support (Table 2).

**Table 2:** Clinical characteristics of adult COVID‐19 patients (*N* = 827 analyzed).

Characteristic	Category	*N*	%
**Clinical Manifestation** (*N* = 748)	Yes	637	77.0
	No	111	13.4
**Comorbidity** (*N* = 789)	Yes (*N* = 384 total)	384	46.4
	No	405	49.0
**Hypertension** (of those with Comorbidity, *N* = 359)	Yes	113	31.5
**Diabetes** (of those with Comorbidity, *N* = 359)	Yes	103	28.7
**Immunocompromised** (of those with Comorbidity, *N* = 359)	Yes	73	20.3
**Ventilatory Support** (*N* = 808)	Yes	522	63.0
	No	286	34.6

*Note:* Percentages for the general characteristics (Manifestation, Comorbidity, Support) are based on the total analyzed sample (*N* = 827). Percentages for specific conditions (Hypertension, Diabetes, Immunocompromised) are based on the *N* of patients with at least one comorbidity (*N* = 359). Missing data for each characteristic are addressed by the *N* value noted in parentheses.

### 3.2 Outcomes and Survival Time

Of the 827 patients analyzed, 139 (17%) experienced the competing event of death, while 516 (62%) achieved recovery. The median time‐to‐event differed significantly between the outcomes: the median time to death was **5 days** (IQR 1–7), while the median time to recovery was **12 days** (IQR 5–16).

Analysis of the death event showed a significant association with age; patients aged ≥ 50 years accounted for 99 of the 139 deaths (71.2%, *p* = 0.007).

### 3.3 Competing Risk Analysis (CRSA) Results

#### 3.3.1 Multivariable Fine‐Gray Model Findings

The multivariable Fine‐Gray model identified several independent factors associated with the sub distribution hazard of death and recovery (Table 3).

**Table 3:** Univariable and multivariable competing risk analysis (Fine‐Gray model) for in‐hospital death and recovery.

Covariate	csHR (95% CI) (Death)	csHR (95% CI) (Recovery)	acsHR (95% CI) (Death)	acsHR (95% CI) (Recovery)
**Age** ≥ **50 years**	2.53 (1.57–4.08)***	0.76 (0.64–0.90)**	2.62 (1.29–5.29)**	0.69 (0.50–0.96)*
**Male (vs. Female)**	1.96 (1.21–3.18)**	1.88 (1.43–2.46)***	0.45 (0.22–0.91)*	0.88 (0.64–1.21)
**Immunocompromised**	2.00 (1.03–3.91)*	1.81 (0.64–5.10)	1.46 (1.08–1.98)*	0.76 (0.62–0.93)**
**Ventilatory support**	1.67 (1.05–2.65)*	1.06 (0.88–1.28)	1.34 (1.12–1.61)**	0.68 (0.44–1.04)

*Note:* *Statistical significance: *p < 0.05, **p < 0.01, ***p < 0.001. The multivariable model was adjusted for age, sex, comorbidity status, and clinical intervention factors. Only variables significant in the multivariable analysis are presented; full model results including non‐significant covariates are available upon request.

Abbreviations: csHR, cause‐specific hazard ratio; acsHR, adjusted cause‐specific hazard ratio; CI, confidence interval.

Regarding the risk of **death (competing event)**, older age (≥ 50 years) was strongly associated with a significantly higher mortality risk (acsHR = 2.62; 95% CI: 1.29, 5.29; *p* < 0.001). Conversely, male sex conferred a significantly lower risk of death (acsHR = 0.45; 95% CI: 0.22, 0.91; *p* = 0.026). Immunocompromised patients showed an increased risk of death (acsHR = 1.46; 95% CI: 1.08, 1.98; *p* = 0.014) and a simultaneously lower probability of recovery (acsHR = 0.76; 95% CI: 0.62, 0.93; *p* = 0.008). The need for ventilatory support was also significantly associated with a higher death risk (acsHR = 1.34; 95% CI: 1.12, 1.61; *p* < 0.001).

### 3.4 Development and Validation of the Asella COVID‐19 Risk Score

**Figure 1:**
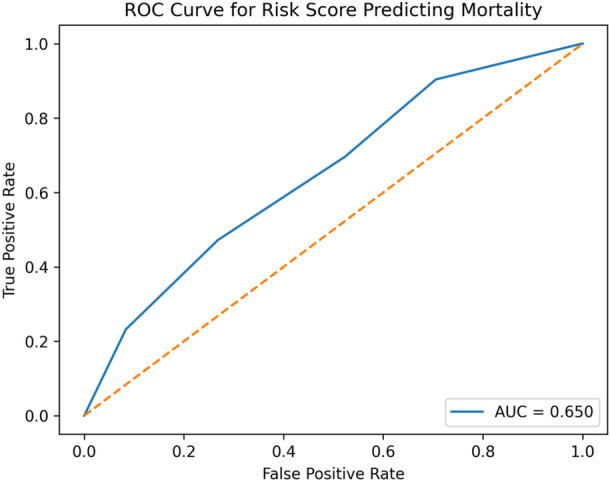
Receiver operating characteristic (ROC) curve for risk score predicting in‐hospital mortality, Asella Ethiopia

To address the lack of context‐specific prognostic tools in our setting, we developed the “Asella COVID‐19 Risk Score” based on readily available clinical parameters (age, comorbidity, and antibiotic use). ROC analysis demonstrated robust discriminatory ability of the Asella Risk Score for predicting in‐hospital mortality (AUC = 0.65). The optimal cutoff value, determined using the Youden Index, was ≥ 4, yielding a sensitivity of 47.2% and a specificity of 73.2%. Furthermore, at this threshold, the score achieved a high negative predictive value (NPV) of 83.5%, indicating that patients scoring below 4 are highly likely to recover (Figure 1).

### 3.5 Cumulative Incidence and Survival Time

**Figure 2:**
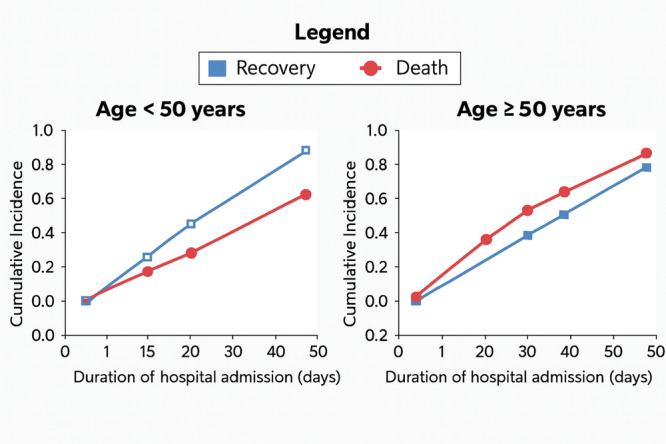
Cumulative incidence function (CIF) curves for recovery and death stratified by age groups.

The median times to outcomes highlighted rapid progression to death among high‐risk patients. The CIF curves confirmed significant differences across age groups. Specifically, the CIF (Figure 2) illustrated a consistently higher death probability and a lower recovery probability among patients aged ≥ 50 years compared to younger patients.

### 3.6 Interpretation Notes

This table demonstrates several key findings:

- **Age** ≥ **50 years:** Significantly increases the hazard of **Death** (acsHR = 2.62) and significantly decreases the hazard of **Recovery** (acsHR = 0.69).
- **Male Sex:** Significantly decreases the hazard of **Death** (acsHR = 0.45).
- **Immunocompromised:** Significantly increases the hazard of **Death** and significantly decreases the hazard of **Recovery**.
- **Ventilatory Support:** Significantly increases the hazard of **Death** (acsHR = 1.34).

## 4 Discussion

### 4.1 Key Findings, Contextual Factors, and Clinical Implications

This study offers a **detailed and rigorous examination** of in‐hospital outcomes for COVID‐19 patients in southeast Ethiopia, utilizing a **competing risk survival analysis (CRSA)** to accurately capture the simultaneous dynamics of recovery and death. This analytical approach provides a more clinically relevant understanding of patient outcomes than traditional survival analyses, which may overestimate survival probabilities in the presence of competing events [23, 25, 29, 30, 31, 32]. Our findings identify advanced age, male gender, and immunocompromised status as the **strongest independent determinants of mortality**, alongside requiring ventilatory support.

Specifically, patients aged **≥ 50 years** faced a **162% higher risk of in‐hospital death** compared to younger patients, underscoring the extreme vulnerability of older adults to severe disease and rapid progression [33, 34]. While global studies often report higher mortality in males [35, 36], our data show that male patients experienced a 55% lower risk of death compared to females. This unexpected finding warrants further investigation into local sociocultural determinants of health‐seeking behaviors and gender‐specific differences in comorbidity prevalence within our population. Concurrently, the **immunocompromised state** emerged as another significant predictor of poor outcomes, increasing the **risk of death by 46%** and simultaneously decreasing the probability of recovery. This finding emphasizes the critical need to **prioritize immunocompromised patients** for early, intensive clinical monitoring and specialized interventions. Similarly, patients requiring ventilatory support, a direct marker of severe illness, exhibited significantly higher mortality, reinforcing the importance of **early recognition and aggressive management** of severe cases [22, 23, 24, 25, 37, 38]. The observed **short median time to death (5 days)** further indicates that COVID‐19 can progress very rapidly, particularly among high‐risk groups, stressing the necessity of **early triage and timely, decisive clinical decision‐making** to reduce mortality. The prognostic landscape for COVID‐19 evolved significantly between the first and second waves, as evidenced by studies on patient stratification and the performance of early prognostic tools [20]. While international models like the 4C Mortality Score and COVID‐GRAM have demonstrated high discriminatory power in high‐resource settings [21], their reliance on specific inflammatory biomarkers (e.g., C‐reactive protein, lymphopenia) often limits their application in resource‐limited clinical settings where such laboratory data may be inconsistently available. To address this, we developed a novel “Asella COVID‐19 Risk Score” based on readily available bedside clinical parameters (Age, Comorbidity, and Antibiotic Use).

This locally derived score achieved an AUC of 0.65, demonstrating robust predictive utility for mortality stratification in our context. Crucially, the score demonstrated a high NPV of 83.5% at the optimal cutoff of ≥ 4. This is a significant clinical strength, as it suggests that patients scoring below this threshold are highly likely to recover. In a high‐burden hospital setting, this provides a powerful triage tool, allowing clinicians to confidently prioritize lower‐intensity care for low‐risk patients while concentrating specialized resources on those at higher risk of adverse outcomes. **The application of this scoring system is summarized in** Table 4.

**Table 4:** Risk stratification based on Asella COVID‐19 risk score.

Risk category	Score range	Clinical implication	Recommended action
Low risk	0–2	High probability of recovery	Routine monitoring; standard care
Moderate risk	3	Intermediate progression risk	Increased surveillance; consider earlier intervention
High risk	≥ 4	High mortality risk	Intensive monitoring; prioritize for specialized/ICU care

*Note:* The Asella COVID‐19 Risk Score was derived from β‐coefficients of the multivariable Fine‐Gray model. Total scores range from 0 to 7 based on age (≥50 years = 2 points), comorbidities (1 point), and antibiotic use (1 point). ICU, Intensive Care Unit. The high‐risk threshold of ≥ 4 was selected based on optimal Youden Index performance to maximize sensitivity and specificity for in‐hospital mortality.

These findings carry **significant implications for clinical practice and health policy**. Hospitals must implement robust early risk stratification protocols, specifically targeting **older, female, and immunocompromised patients** to ensure they receive timely and aggressive interventions. Resource allocation, including the deployment of critical care beds and ventilatory support, should be guided by these identified risk factors to maximize survival gains. Furthermore, the study validates the utility of competing risk models in accurately characterizing complex, multi‐state outcomes in hospitalized COVID‐19 populations, particularly in resource‐limited settings [22, 23, 24]. Despite the strengths of a multicenter approach and the use of CRSA, the study has limitations inherent to its **retrospective design**. Potential **selection and information biases**, as well as the presence of unmeasured confounders (e.g., precise timing of interventions/symptom onset), may influence the outcomes. However, the comprehensive data collection and rigorous analytical approach mitigate some of these concerns.

## 5 Conclusion

This study conclusively demonstrates that **older age, female gender, and immunocompromised status** are significant independent risk factors that substantially increase the risk of in‐hospital mortality among COVID‐19 patients in southeast Ethiopia. The “Asella COVID‐19 Risk Score” serves as a validated, clinically accessible tool for rapid bedside stratification. By enabling early identification of high‐risk patients, this tool provides a practical framework to improve clinical outcomes and optimize resource utilization, even in settings with limited diagnostic infrastructure.

## 6 Recommendations

Based on our findings, we propose the following actions:

1. **Clinical Implementation:** Hospitals in resource‐limited settings should adopt the “Asella COVID‐19 Risk Score” as a standard triage tool upon patient admission to facilitate immediate risk stratification.
2. **Resource Allocation:** Patients identified as “High Risk” (score ≥ 4) by the tool should be prioritized for intensive care monitoring and early clinical intervention.
3. **Future Research:** We recommend prospective validation of the Asella COVID‐19 Risk Score across diverse healthcare settings in Ethiopia to further refine its predictive accuracy and utility.
4. **Targeted Policy:** Health authorities should develop gender‐sensitive clinical protocols to address the observed higher mortality risk among female patients in the region.

## Author Contributions

**Addis Wordofa, Ayalneh Demissie**, and **Abdurehman Tune:** conceptualization, data collection, writing – original draft. **Ayalneh Demissie:** methodology, supervision, writing – review and editing. **Abdurehman Kalu, Abdurehman Tune, Mohammed Suleiman Obsa, Abay Kibret, Zerihun Abera**, and **Yonas Mulugeta:** data curation, validation, review and editing.

## Ethics Statement

This study was approved by Arsi University's Health Research Ethical Review Committee (Accreditation number: ERC No A/CHS/RC/75/2023). Permission for data collection was obtained from participating treatment centers. All patient data were anonymized and de‐identified prior to extraction. No direct patient contact occurred, and informed consent was waived due to the use of retrospective records.

## Conflicts of Interest

The authors declare no conflicts of interest

## Transparency Statement

The lead author, Ayalneh Demissie, affirms that this manuscript is an honest, accurate, and transparent account of the study being reported; that no important aspects of the study have been omitted; and that any discrepancies from the study as planned (and, if relevant, registered) have been explained.

## Data Availability

The datasets generated and analyzed during the current study are provided within the manuscript and as supporting material.
